# Modified EPOCH for high‐risk non‐Hodgkin lymphoma in sub‐Saharan Africa

**DOI:** 10.1002/cam4.2631

**Published:** 2019-11-09

**Authors:** Takondwa Zuze, Grace K. Ellis, Edwards Kasonkanji, Bongani Kaimila, Richard Nyasosela, Ruth Nyirenda, Tamiwe Tomoka, Maurice Mulenga, Maria Chikasema, Blessings Tewete, Asekanadziwa Mtangwanika, Sarah Chiyoyola, Fred Chimzimu, Coxcilly Kampani, Wilberforce Mhango, Simon Nicholas, Cara Randall, Nathan D. Montgomery, George Fedoriw, Katherine D. Westmoreland, Matthew S. Painschab, Satish Gopal

**Affiliations:** ^1^ UNC Project‐Malawi Lilongwe Malawi; ^2^ University of Malawi College of Medicine Blantyre Malawi; ^3^ Kamuzu Central Hospital Lilongwe Malawi; ^4^ University of North Carolina Chapel Hill NC USA

**Keywords:** global health, modified EPOCH, non‐Hodgkin lymphoma, sub‐Saharan Africa

## Abstract

Aggressive non‐Hodgkin lymphoma (NHL) is among the most common cancers in sub‐Saharan Africa (SSA), where CHOP is standard treatment and outcomes are poor. To address this, we treated 17 newly diagnosed adult patients in Malawi with Burkitt (n = 8), plasmablastic (n = 8), and primary effusion lymphoma (n = 1) with a modified EPOCH regimen between 2016 and 2019. Twelve patients (71%) were male and the median age was 40 years (range 16‐63). Eleven (65%) were HIV infected, median CD4 count was 218 cells/µL (range 9‐460), and nine (82%) had suppressed HIV RNA < 400 copies/mL. Patients received a median of six cycles (range 2‐8) and median follow‐up was 14 months (range 2‐34) among patients still alive. Grade 3/4 neutropenia was observed in 26% of cycles and in 65% of patients. Sixteen (94%) responded to EPOCH and 10 (59%) achieved a complete response. One‐year overall survival (OS) was 62% (95% confidence interval [CI], 42%‐91%). Five patients (29%) died from progressive NHL and three (18%) from treatment‐related complications. These data suggest EPOCH with setting‐appropriate modifications may be a practical, safe, and effective option for improving high‐risk NHL outcomes in Malawi and comparable settings, which deserves further prospective evaluation.

## Graphical abstract

We evaluated the safety and efficacy of EPOCH, with modifications as required for local administration, for patients with high‐risk NHL in Malawi. We treated 17 newly diagnosed adult patients in Malawi with Burkitt (n = 8), plasmablastic (n = 8), and primary effusion lymphoma (n = 1) with a modified EPOCH regimen between 2016 and 2019. Our experience suggests that EPOCH with setting‐appropriate modifications may be a practical, safe, and effective option for improving high‐risk NHL outcomes in Malawi and comparable settings, which deserves further prospective evaluation.

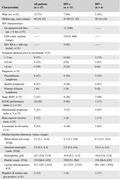

## 1 INTRODUCTION

Aggressive non‐Hodgkin lymphoma (NHL) subtypes predominate in sub‐Saharan Africa (SSA),^1^ reflecting a young population and high prevalence of HIV and oncogenic viruses, including Epstein‐Barr virus (EBV) and Kaposi sarcoma‐associated herpesvirus (KSHV).^2^

Diffuse large B‐cell lymphoma (DLBCL) is the most common aggressive NHL worldwide,^1^ for which rituximab plus CHOP (cyclophosphamide, doxorubicin, vincristine, prednisone) is the international standard of care.^3^, ^4^ Treatment intensification has not improved DLBCL outcomes, although individualized approaches based on molecular characterization are being investigated. For other aggressive NHLs, like Burkitt lymphoma (BL), CHOP rarely leads to durable disease control. In high‐income countries, BL has historically been treated with intensive regimens modeled after pediatric leukemia therapies, associated with significant toxicities and requiring high levels of support.^5^, ^6^

Lower‐intensity infusional regimens, notably dose‐adjusted EPOCH (etoposide, prednisolone, vincristine, cyclophosphamide, doxorubicin), have been developed as an alternative to reduce treatment‐related toxicities while maintaining excellent outcomes.^7^, ^8^ Cytotoxicity in proliferative lymphoma cells may be enhanced by prolonged chemotherapy exposure, and EPOCH has gained acceptance as a standard option for BL, plasmablastic lymphoma (PBL), and primary effusion lymphoma (PEL) in high‐income countries.^8^, ^9^, ^10^, ^11^

In SSA, resource‐limited health systems, high opportunistic infection burden, and frequent comorbid HIV and/or malnutrition make high‐intensity cytotoxic chemotherapy challenging.^12^, ^13^ We hypothesized that EPOCH, with modifications as required for local administration, might be safe and effective for high‐risk NHL at a national teaching hospital in Malawi, particularly in light of historically poor outcomes using less intense strategies.^14^, ^15^, ^16^, ^17^ To our knowledge, no prior reports describe EPOCH application in a low‐income country in SSA, where aggressive NHL subtypes are common and therapeutic options are severely limited.

## 2 Methods

In August 2016, based on poor outcomes for patients receiving CHOP, institutional practice was changed such that newly diagnosed adult patients at Kamuzu Central Hospital (KCH) with BL, PBL, and PEL received modified EPOCH. All patients with newly diagnosed lymphoproliferative disorders were enrolled in the KCH Lymphoma Study prospective cohort previously described.^18^ All diagnoses were pathologically confirmed using tissue biopsies and immunohistochemistry, including stains performed on site for CD3, CD20, CD45, CD138, BCL2, TDT, PAX5, and Ki67, interpreted during real‐time weekly telepathology conferences involving two to four United States and Malawian pathologists who rendered a consensus opinion, and subsequent secondary review by United States hematopathologists.^19^ Fluorescence in situ hybridization capabilities are not present in Malawi, and were typically not done subsequently in the United States due to limited diagnostic specimens remaining after initial real‐time work‐up. Patients were staged using physical examination, chest radiography, abdominal ultrasound, unilateral bone marrow biopsy, and cerebrospinal fluid cytology, as previously described.^18^

Patients received modified EPOCH as per the United States National Cancer Institute (NCI) protocol beginning at dose level +1 (etoposide 50 mg/m^2^, vincristine 0.4 mg/m^2^, and doxorubicin 10 mg/m^2^ days 1‐4; prednisone 60 mg/m^2^ days 1‐5; cyclophosphamide 750 mg/m^2^ day 1). Treatment was administered using peripheral, rather than central, venous catheters. Limited clinic and hospital infrastructure were felt to prohibit safe administration of infusional chemotherapy outside normal cancer clinic operating hours, especially overnight when nursing support was scarce and risk of extravasation or other adverse events was felt to be excessively high. Hence, 24‐hour doses of etoposide, vincristine, and doxorubicin were administered over 8 hours on four successive days in clinic. For tumor lysis syndrome prevention, patients typically received allopurinol plus prephase treatment with prednisone for 5‐7 days, then CVP (cyclophosphamide, vincristine, prednisone), followed by EPOCH initiation 1 week later. Patients at high risk for leptomeningeal involvement, including all BL patients and PBL patients with craniofacial involvement, had baseline cerebrospinal fluid cytology performed and received intrathecal methotrexate and hydrocortisone with each cycle. Treatment cycles were administered every 21 days and continued until progression, death, or completion of six cycles.

Based on adaptation of the NCI protocol as required for local implementation, chemotherapy doses were adjusted to achieve an absolute neutrophil count (ANC) of 1.0‐1.5 × 10^3^/µL on day 1 of the next scheduled cycle, with dose escalation if ANC was >1.5 × 10^3^/µL on day 1 of the next scheduled cycle. Patients could not generally return for twice‐weekly blood counts per NCI protocol recommendations, due to long travel distances with high transportation costs. Rituximab and hematopoietic growth factors were not available in the Malawian public sector during this period. All patients received prophylactic ciprofloxacin on days 7‐21 of each cycle. HIV‐infected patients additionally received prophylactic cotrimoxazole and fluconazole continuously throughout chemotherapy, as well as concurrent antiretroviral therapy (ART) with tenofovir‐lamivudine‐efavirenz, the recommended first‐line regimen in Malawi during the study period.

Cohort characteristics were summarized using simple descriptive statistics. Response was assessed according to the standard international criteria,^20^ using physical examination, chest radiography, and abdominal ultrasound. Adverse events were reported using NCI Common Terminology Criteria for Adverse Events version 4.0. Responses were assessed as previously described.^18^ ANC curves were generated by averaging available ANC measurements on day 1 of each cycle. Patients were followed from enrollment until death or administrative censoring on 30 June 2019. Kaplan‐Meier methods were used to estimate overall survival (OS) and progression‐free survival (PFS) from the date of cohort enrollment. Analyses were conducted using R 3.5.2 (New York, New York). All participants gave written informed consent. This study was approved by the UNC Institutional Review Board and Malawi National Health Sciences Research Committee.

## 3 RESULTS

Between August 2016 and November 2018, 21 patients with BL, PBL, and PEL were enrolled. During this period, four eligible patients with BL, PBL, or PEL diagnoses did not receive modified EPOCH due to death before treatment initiation (n = 1), poor performance status (n = 1), or patient reluctance to spend four successive days in clinic per cycle (n = 2). Seventeen patients were thus treated with modified EPOCH (8 BL, 8 PBL, and 1 PEL). Twelve patients (71%) were male and the median age was 40 years (range 16‐63) (Table 1). Eleven patients (65%) were HIV infected, among whom median CD4 count was 218 cells/µL (range 9‐460), and nine (82%) had suppressed HIV RNA < 400 copies/mL. Although the testing of tumor specimens for EBV was not available onsite in Malawi during the study period, five (29%) were tested in the United States, of which four (80%) were EBV positive (1/1 BL, 3/4 PBL, 0/0 PEL).

**Table 1:** Characteristics of patients receiving modified EPOCH in Lilongwe, Malawi

Characteristic	All patients (n = 17)	HIV+ (n = 11)	HIV− (n = 6)
Male sex, n (%)	12 (71)	7 (64)	5 (83)
Median age, years (range)	40 [16, 63]	41.00 [21, 55]	20 [16, 63]
HIV characteristics			
On antiretroviral therapy > 6 mo, n (%)	—	11 (100)	—
CD4 count, median (range)	—	218 [9, 460]	—
HIV RNA < 400 copies/mL, n (%)	—	9 (82)	—
Symptom duration prior to enrollment, n (%)			
1‐3 mo	8 (47)	6 (54)	2 (33)
4‐6 mo	4 (23)	0 (0)	4 (67)
>6 mo	5 (29)	5 (45)	0 (0)
Diagnosis, n (%)			
Plasmablastic lymphoma	8 (47)	6 (54)	2 (33)
Burkitt lymphoma	8 (47)	4 (36)	4 (67)
Primary effusion lymphoma	1 (6)	1 (9)	0 (0)
Stage III/IV, n (%)	7 (41)	4 (36)	3 (50)
ECOG performance status ≥ 2, n (%)	10 (59)	9 (82)	1 (17)
International prognostic index ≥ 3, n (%)	7 (41)	3 (27)	4 (67)
Bone marrow involvement, n (%)	2 (12)	1 (9)	1 (17)
Extranodal involvement, n (%)	9 (53)	4 (36)	5 (83)
Median baseline laboratory values, (range)			
White blood cell count, 10^3^/µL	5.2 [2.1, 14.4]	5.2 [2.1, 9.8]	6.3 [3.5, 14.4]
Absolute neutrophil count, 10^3^/µL	2.9 [0.5, 8.4]	2.9 [0.5, 6.6]	3.0 [1.4, 8.4]
Hemoglobin, g/dL	10.7 [5.8, 12.9]	10.0 [6.2, 12.4]	10.8 [5.8, 12.9]
Platelet count, 10^3^/uL	339 [66.0, 635]	339[151, 584]	334 [66.0, 635]
Lactate dehydrogenase, IU/L*	417 [165, 2,522]	412 [233, 2,522]	881 [165, 1,870]
Hepatitis B surface antigen positive, n (%)	2 (12)	1 (9)	1 (17)

* Laboratory upper limit of normal 250 IU/L.

**Figure 1:**
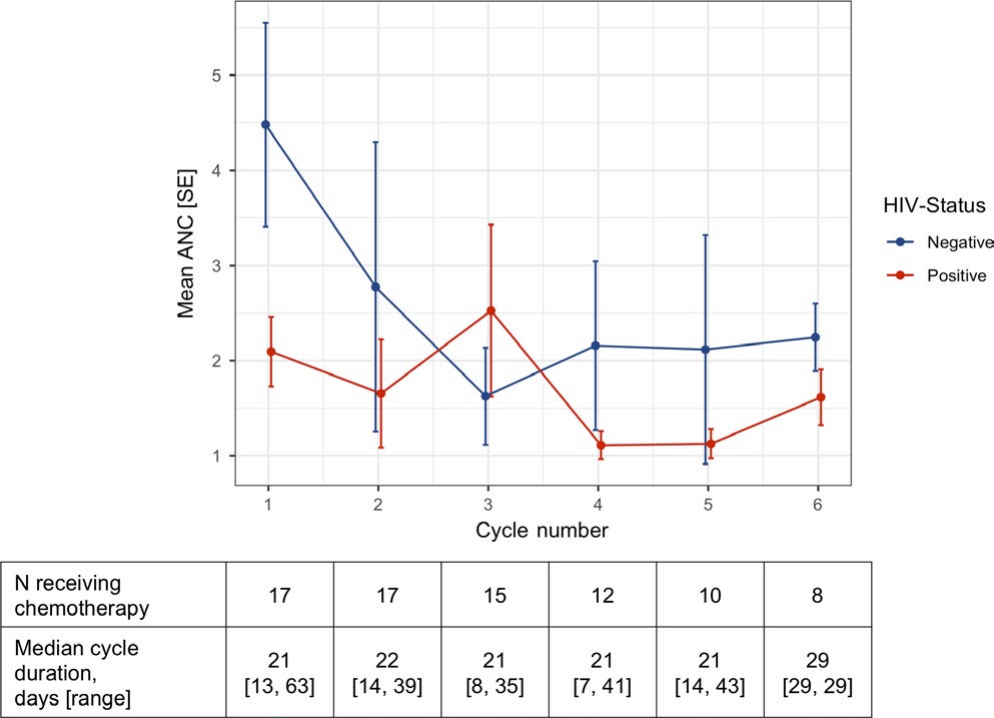
Mean absolute neutrophil count during modified EPOCH in Lilongwe, Malawi

Patients received a median six EPOCH cycles (range 2‐8), with median NCI dose level +1 (range −2 to +4) per cycle and without major differences by HIV status (Table 2). No patients abandoned treatment, and six (35%) of patients completed all EPOCH cycles at NCI dose level +1 or greater. Mean ANC trajectories for the overall cohort stratified by HIV status are shown in Figure 1. As anticipated, mean ANC during treatment was lower in HIV‐infected patients. Grade 3/4 neutropenia, based on planned day 1 blood counts for each cycle without interim assessments, occurred in 65% of patients and in 26% of cycles. Deaths are described separately below, but no documented grade 3/4 non‐hematologic adverse events were observed, including no chemotherapy extravasation events.

**Table 2:** Treatment course and toxicities among patients receiving modified EPOCH in Lilongwe, Malawi

	Cycles			Patients			
	All (n = 83)	HIV+ (n = 50)	HIV− (n = 33)		All (n = 17)	HIV+ (n = 11)	HIV− (n = 6)
Median NCI protocol dose level (range)	+1 (−2 to +4)	+1 (−2 to +2)	+1 (−2 to +4)		—	—	—
Neutropenia < 1.0 × 10^3^/µL, n (%)	22 (27)	14 (29)	8 (24)		11 (65)	7 (64)	4 (67)
Neutropenia < 0.5 × 10^3^/µL, n (%)	4 (5)	2 (4)	2 (6)		3 (18)	2 (18)	1 (17)
Thrombocytopenia < 50 × 10^3^/µL, n (%)	2 (2)	0 (0)	2 (6)		2 (12)	0 (0)	2 (33)
Febrile neutropenia	1 (1)	0 (0)	1 (3)		1 (6)	0 (0)	1 (17)
Non‐hematologic grade 3/4 adverse events, n (%)	0 (0)	0 (0)	0 (0)		0 (0)	0 (0)	0 (0)

Abbreviation: NCI, National Cancer Institute.

**Figure 2:**
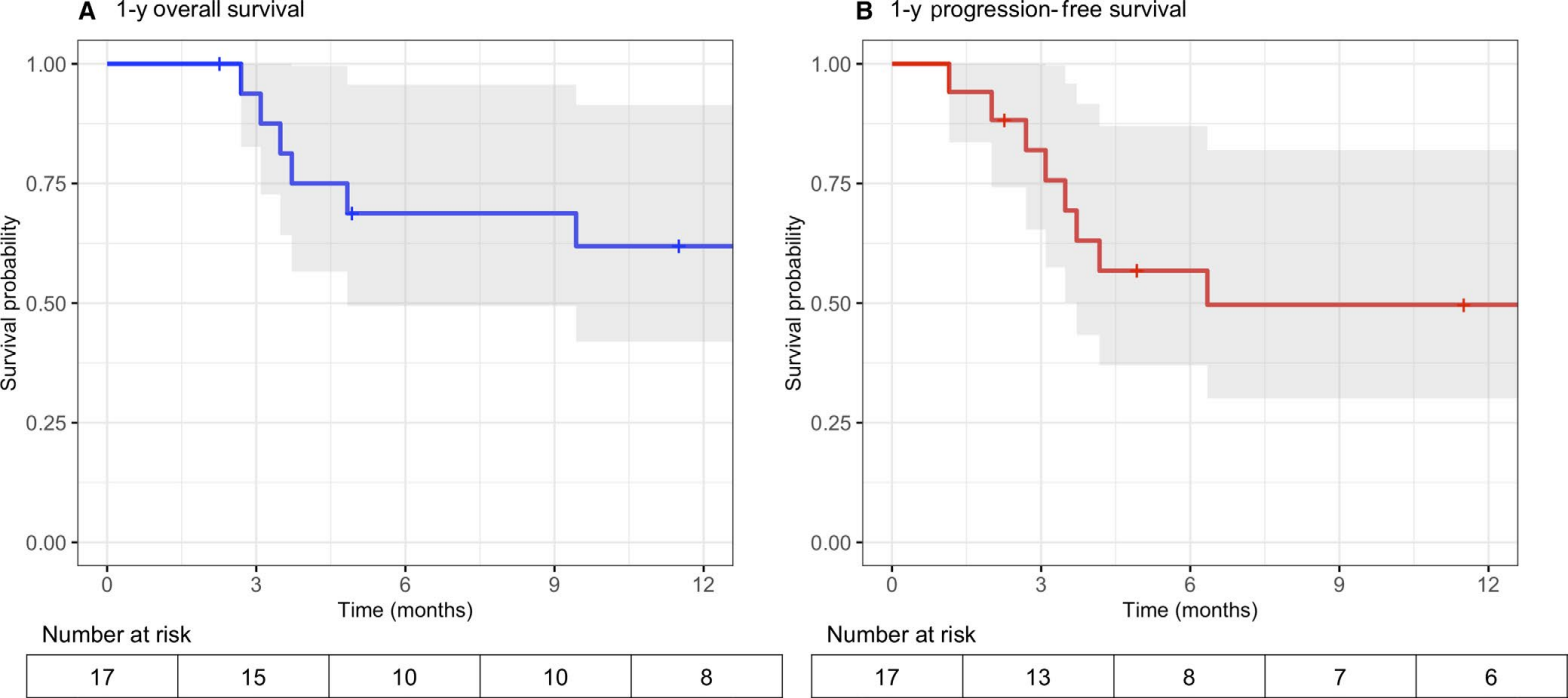
A, Overall survival. B, Progression‐free survival. Kaplan‐Meier survival estimates with 95% confidence intervals among patients receiving modified EPOCH in Lilongwe, Malawi

As of 30 June 2019, vital and disease status was known for all 17 patients, with median follow‐up 14.4 months (range 2.3‐33.9) among patients still alive (Table 3). Ten patients (59%) achieved complete response (3/8 PBL, 7/8 BL, 0/1 PEL), six (35%) achieved partial response, and one had refractory disease. One‐year OS was 62% (95% confidence interval [CI], 42%‐91%), and 1‐year PFS was 50% (95% CI, 30%‐82%) (Figure 2). Of eight deaths, five were from progressive lymphoma and three from EPOCH complications, with 18% treatment‐related mortality. Presumed treatment‐related deaths occurred shortly after receiving an EPOCH cycle, and patients presented to local health centers with sepsis‐like symptoms, such as fever, diarrhea, and vomiting. Due to limited diagnostic capabilities at these health centers, a more specific cause of death could not be identified. For these three patients, administered EPOCH dose level ranges were +1 to +1, +1 to +2, and +1 to +4, respectively.

**Table 3:** Outcomes among patients receiving modified EPOCH in Lilongwe, Malawi

	All patients (n = 17)	HIV+ (n = 11)	HIV− (n = 6)	PBL (n = 8)	BL (n = 8)
Complete response, n (%)	10 (59)	7 (64)	3 (50)	3 (37)	7 (87)
Follow‐up months among patients still alive, median (range)	14.4 (2.3, 33.9)	12.9 (2.3, 29.3)	17.3 (13.9, 33.9)	15.6 (2.3, 29.3)	14.4 (4.9, 33.9)
1‐y overall survival (95% CI)	62% (42%‐91%)	58% (34%‐100%)	67% (38‐100)	43% (18%‐100%)	75% (50%‐100%)
1‐y progression‐free survival (95% CI)	50% (30%‐82%)	48% (25%‐94%)	50% (22%‐100%)	29% (9%‐93%)	75% (50%‐100%)
Deaths, n (%)	8 (47)	5 (45)	3 (50)	4 (50)	3 (37)
Treatment‐related deaths, n (%)	3 (18)	2 (18)	1 (17)	2 (25)	1 (12)

## 4 DISCUSSION

International standards of care for high‐risk NHL are associated with significant toxicities and require robust support, making them impractical for many SSA settings.^12^, ^13^ EPOCH is a more recent strategy in high‐income countries to address some of these challenges.^8^, ^9^, ^10^, ^11^ We provide the first detailed prospective data describing specific application of the EPOCH regimen in a low‐income country in SSA, with modifications as required by the setting. We previously reported possibly improved outcomes for higher‐intensity treatment among adolescents and adults with BL in Malawi, using EPOCH or high‐dose methotrexate‐based treatment vs CHOP.^15^ The current paper includes the four patients with BL treated with EPOCH reported earlier, but with more detailed data regarding EPOCH implementation, dose intensity, toxicity, and efficacy in a larger cohort of patients, to inform potential application of this approach in contexts similar to Malawi. Modified EPOCH appeared to be feasible and effective in a small cohort of HIV‐positive and HIV‐negative patients with BL, PBL, and PEL in Malawi, even under public sector conditions and without hematopoietic growth factor support, although treatment‐related mortality was significant. Responses and outcomes in patients receiving EPOCH may be better than in patients historically receiving CHOP, including 23% overall survival at 2 years for adolescents and adults treated with CHOP in Malawi.^14^, ^15^ Neutropenia was common, which is an intended consequence of EPOCH administration, making dose escalation challenging for most patients. Despite standardized anti‐infective prophylaxis to mitigate neutropenia risks, three patients died from treatment‐related complications. Treatment‐related mortality, however, did not markedly differ from experience with CHOP in our setting.^14^, ^15^, ^21^

To our knowledge, this is the first study describing attempted EPOCH application for high‐risk NHL in SSA, primarily in patients with BL or PBL. Study strengths include prospective design, protocol‐guided treatment, standardized clinical and toxicity assessment, and complete vital status and response assessment. Study limitations are small cohort size enrolled at a single institution with limited follow‐up, and real‐world resource and logistical constraints imposed by the health care system in one of the economically poorest countries in the world. In conclusion, however, our experience suggests infusional cytotoxic approaches, with setting‐appropriate modification, may be a partial solution to improve outcomes for aggressive NHL subtypes in SSA, which have historically done very poorly using conventional approaches. EPOCH may be a particularly valuable strategy in SSA as health systems improve and provide better supportive care infrastructure, including routine availability of hematopoietic growth factors, to allow administration, monitoring, and dose intensity that better approximate EPOCH as used in high‐income countries. Application and adaption of the EPOCH regimen therefore deserves further multicenter evaluation specifically in SSA to address this major ongoing clinical need.

## CONFLICT OF INTEREST

The authors declare no competing financial interests.

## AUTHORSHIP

Contribution: TZ, GKE, and SG designed the study, performed the analyses, and wrote the paper. TZ, EK, BK, RN, RN, MC, BT, AM, SC, WM, SN, KDW, MSP, and SG provided clinical care. TT, MM, FC, CK, CR, NDM, and YF reviewed all pathology specimens.

## Data Availability

The data that support the findings of this study are available on request from the corresponding author. The data are not publicly available due to privacy or ethical restrictions.

## References

[1] Perry AM , Diebold J , Nathwani BN , et al. Non‐Hodgkin lymphoma in the developing world: review of 4539 cases from the International Non‐Hodgkin Lymphoma Classification Project. Haematologica. 2016;101(10):1244‐1250. 27354024 10.3324/haematol.2016.148809 PMC5046654

[2] Plummer M , de Martel C , Vignat J , Ferlay J , Bray F , Franceschi S . Global burden of cancers attributable to infections in 2012: a synthetic analysis. Lancet Glob Heal. 2016;4(9):e609‐e616. 10.1016/S2214-109X(16)30143-7 27470177

[3] Coiffier B , Lepage E , Brière J , et al. CHOP chemotherapy plus rituximab compared with CHOP alone in elderly patients with diffuse large‐B‐cell lymphoma. N Engl J Med. 2002;346(4):235‐242. 11807147 10.1056/NEJMoa011795

[4] Pfreundschuh M , Kuhnt E , Trümper L , et al. CHOP‐like chemotherapy with or without rituximab in young patients with good‐prognosis diffuse large‐B‐cell lymphoma: 6‐year results of an open‐label randomised study of the MabThera International Trial (MInT) Group. Lancet Oncol. 2011;12(11):1013‐1022. 21940214 10.1016/S1470-2045(11)70235-2

[5] Patte C , Auperin A , Michon J , et al. The Société Française d'Oncologie Pédiatrique LMB89 protocol: highly effective multiagent chemotherapy tailored to the tumor burden and initial response in 561 unselected children with B‐cell lymphomas and L3 leukemia. Blood. 2001;97(11):3370‐3379. 11369626 10.1182/blood.v97.11.3370

[6] Mead GM , Sydes MR , Walewski J , et al. An international evaluation of CODOX‐M and CODOX‐M alternating with IVAC in adult Burkitt's lymphoma: results of United Kingdom Lymphoma Group LY06 study. Ann Oncol Off J Eur Soc Med Oncol. 2002;13(8):1264‐1274. Accessed March 28, 2019. 10.1093/annonc/mdf253 12181251

[7] Wilson WH , Grossbard ML , Pittaluga S , et al. Dose‐adjusted EPOCH chemotherapy for untreated large B‐cell lymphomas: a pharmacodynamic approach with high efficacy. Blood. 2002;99(8):2685‐2693. 11929754 10.1182/blood.v99.8.2685

[8] Dunleavy K , Pittaluga S , Shovlin M , et al. Low‐intensity therapy in adults with Burkitt's lymphoma. N Engl J Med. 2013;369(20):1915‐1925. 24224624 10.1056/NEJMoa1308392 PMC3901044

[9] Castillo JJ , Bibas M , Miranda RN . The biology and treatment of plasmablastic lymphoma. Blood. 2015;125(15):2323‐2330. 25636338 10.1182/blood-2014-10-567479

[10] Lurain K , Polizzotto MN , Aleman K , et al. Viral, immunologic, and clinical features of primary effusion lymphoma. Blood. 2019;133(16):1753‐1761. 30782610 10.1182/blood-2019-01-893339 PMC6473499

[11] Dunleavy K , Roschewski M , Abramson JS , et al. Risk‐adapted therapy in adults with burkitt lymphoma: updated results of a multicenter prospective phase II study of DA‐EPOCH‐R. Hematol Oncol. 2017;35(S2):133‐134.

[12] Gopal S , Wood WA , Lee SJ , et al. Meeting the challenge of hematologic malignancies in sub‐Saharan Africa. Blood. 2012;119(22):5078‐5087. 22461494 10.1182/blood-2012-02-387092 PMC4507039

[13] Gopal S , Gross TG . How I treat Burkitt lymphoma in children, adolescents, and young adults in sub‐Saharan Africa. Blood. 2018;132(3):254‐263. 29769263 10.1182/blood-2018-04-844472 PMC6053950

[14] Zuze T , Painschab MS , Seguin R , et al. Plasmablastic lymphoma in Malawi. Infect Agent Cancer. 2018;13(1):22. 29988350 10.1186/s13027-018-0195-4 PMC6022505

[15] Painschab MS , Westmoreland KD , Kasonkanji E , et al. Prospective study of Burkitt lymphoma treatment in adolescents and adults in Malawi. Blood Adv. 2019;3(4):612‐620. 30796065 10.1182/bloodadvances.2018029199 PMC6391663

[16] Westmoreland KD , Montgomery ND , Stanley CC , et al. Plasma Epstein‐Barr virus DNA for pediatric Burkitt lymphoma diagnosis, prognosis and response assessment in Malawi. Int J cancer. 2017;140(11):2509‐2516. 28268254 10.1002/ijc.30682 PMC5386821

[17] Stanley CC , Westmoreland KD , Heimlich BJ , et al. Outcomes for paediatric Burkitt lymphoma treated with anthracycline‐based therapy in Malawi. Br J Haematol. 2016;173(5):705‐712. 26914979 10.1111/bjh.13986 PMC4884132

[18] Gopal S , Fedoriw Y , Kaimila B , et al. Chemotherapy for aggressive non‐hodgkin lymphoma with and without HIV in the antiretroviral therapy era in Malawi. PLoS ONE ONE. 2016;11(3):e0150445. 10.1371/journal.pone.0150445 PMC4775030 26934054

[19] Montgomery ND , Liomba NG , Kampani C , et al. Accurate real‐time diagnosis of lymphoproliferative disorders in malawi through clinicopathologic teleconferences. Am J Clin Pathol. 2016;146(4):423‐430. 27594430 10.1093/ajcp/aqw118 PMC5040876

[20] Cheson BD , Fisher RI , Barrington SF , et al. Recommendations for initial evaluation, staging, and response assessment of Hodgkin and non‐Hodgkin lymphoma: the Lugano classification. J Clin Oncol. 2014;32(27):3059‐3068. 25113753 10.1200/JCO.2013.54.8800 PMC4979083

[21] Painschab MS , Kasonkanji E , Zuze T , et al. Mature outcomes and prognostic indices in diffuse large B‐cell lymphoma in Malawi: a prospective cohort. Br J Haematol. 2019;184(3):364‐372. 30450671 10.1111/bjh.15625 PMC6340743

